# Gingival Inflammation Modulates NLRP3 Inflammasome Signalling in Peripheral Blood Mononuclear Cells of PCOS Patients: A Case-Control Study

**DOI:** 10.3390/antiox14050507

**Published:** 2025-04-24

**Authors:** Cecilia Fabiana Márquez-Arrico, María Pelechá-Salvador, Meylin Fernández-Reyes, Francisco Javier Silvestre, Laura Perea-Galera, Jonathan Hermenejildo, Zaida Abad-Jiménez, Javier Silvestre-Rangil, Carlos Morillas, Víctor M. Víctor, Sandra López-Domènech, Milagros Rocha

**Affiliations:** 1 Department of Stomatology, University of Valencia, 46010 Valencia, Spain; cecilia.marquez@uv.es (C.F.M.-A.); francisco.silvestre@uv.es (F.J.S.); javier.silvestre@uv.es (J.S.-R.); 2 Department of Endocrinology and Nutrition, University Hospital Doctor Peset, Foundation for the Promotion of Health and Biomedical Research, 46017 Valencia, Spain; maria.pelecha@fisabio.es (M.P.-S.); meylin.fernandez@fisabio.es (M.F.-R.); laura.perea@fisabio.es (L.P.-G.); jonathan.hermnejildo@fisabio.es (J.H.); zmabad@fisabio.es (Z.A.-J.); carlos.morillas@uv.es (C.M.); 3 Department of Stomatology, University Hospital Doctor Peset, Foundation for the Promotion of Health and Biomedical Research, 46017 Valencia, Spain; 4 Department of Physiology, Biomedical Research Institute Valencia (INCLIVA), University of Valencia, 46010 Valencia, Spain; victor.victor@uv.es; 5 National Network of Biomedical Research on Hepatic and Digestive Diseases (CIBEREHD), Institute of Health Carlos III, 28029 Madrid, Spain

**Keywords:** inflammasome, NLRP3 protein, PBMCs, gingivitis, polycystic ovary syndrome, periodontal disease

## Abstract

Polycystic ovary syndrome (PCOS) is a complex condition associated with chronic inflammation and oxidative stress and is often linked to periodontal diseases. This study aimed to determine whether gingivitis modulates the NLRP3 inflammasome in peripheral blood mononuclear cells (PBMCs) from women with PCOS. Following a case-control design, 104 women were divided into three groups: controls (n = 36), PCOS without gingivitis (PCOS, n = 44) and PCOS with gingivitis (PCOS+, n = 24). Periodontal parameters, proinflammatory regulators (NFκB p65, JNK), NLRP3 components (NLRP3, ASC, procaspase-1, caspase-1) and oxidative stress markers (superoxide, *NRF2*, *GCLC* and *GSR*) were determined. The PCOS+ group presented elevated values for bleeding on probing (BOP) and plaque and calculus indices, both of which were associated with increased protein levels of NFκB p65 and JNK, thus indicating NLRP3 inflammasome priming. Higher protein levels of NLRP3, ASC, procaspase-1 and caspase-1 in the PCOS+ group confirmed that priming had occurred, suggesting an engagement in assembly. When potential assembly signals of inflammasome were evaluated, the patients with PCOS generally presented enhanced total superoxide and an impaired antioxidant response (*NRF2, GCLC* and *GSR*). Moreover, BOP was independently associated with JNK, ASC and procaspase-1. These findings suggest that gingival inflammation modulates the innate immune response in leukocytes of women with PCOS via the NLRP3 inflammasome pathway, which is regulated by proinflammatory factors and oxidative damage.

## 1. Introduction

Polycystic ovary syndrome (PCOS) is a complex endocrine disease affecting 5.5–19.9% of reproductive-aged women worldwide [1,2]. This gynaecological condition is characterised by clinical/biochemical hyperandrogenism, ovulatory dysfunction (including oligo-ovulation and anovulation) and multiple ovarian cysts [3]. PCOS can lead to obesity, infertility, insulin resistance (IR), hirsutism, acne and amenorrhea [4]. Moreover, evidence links the oxidative stress status seen in patients and characterised by pro-oxidant mediators [4,5] to the promotion of chronic low-grade inflammation [6]. As a whole, women with PCOS are at an increased risk of cardiometabolic outcomes, such as type 2 diabetes or cardiovascular disease [7]. Physiological fluctuations in sex hormones, such as those that occur during the premenopausal and postmenopausal periods or pregnancy, can influence the characteristics of the gingival epithelium and crevicular fluid, thus increasing susceptibility to periodontal diseases (PD) [8]. Similarly, growing evidence indicates that women with PCOS present more frequent alterations in periodontal parameters compared to healthy individuals, suggesting a link between PCOS and PD, including gingivitis and periodontitis [9,10,11,12]. Periodontitis is a chronic multifactorial inflammatory disease associated with dysbiotic plaque biofilms. Bacterial infections and host immune responses that occur as a result of periodontitis create a distinct microenvironment, which affects periodontal homeostasis, leading to the progressive destruction of the tooth-supporting structures, including the alveolar bone, gingiva and periodontal ligament [13]. Genetic, environmental and systemic factors have been implicated in its development, and it has been associated with conditions such as diabetes and cardiovascular diseases. The relation between PCOS and PD may stem from hormonal imbalances in PCOS, which contribute to gingival inflammation [8], while IR, systemic inflammation and oxidative stress further promote a proinflammatory oral environment, increasing the risk of PD progression [11,14]. Indeed, IR, which is recurrent in women with PCOS, has been associated with different parameters of periodontal infection [14,15,16].

Gingivitis is the most prevalent form of periodontal disease, affecting the gingival epithelium and underlying connective tissue. It is characterised by symptoms such as tissue redness, tenderness, swelling and bleeding upon gentle probing. Histopathologically, gingivitis involves alterations including elongation of the rete ridges, dilatation of blood vessels, destruction of collagen fibres and a progressive inflammatory infiltrate. The condition is caused primarily by the accumulation of dental plaque, which triggers an inflammatory response in the host tissues [17]. This promotes bacterial migration to the surrounding periodontal pockets, leading to the recruitment of leukocytes from the bloodstream to the infection site, thereby increasing local oxidative stress and often progressing to irreversible tissue damage and tooth loss [18,19]. Despite being a localised condition, gingivitis can lead to more severe periodontal diseases if left untreated, for example, periodontitis [18,20]. Traditional treatment focuses on reducing the oral bacterial load through improved oral hygiene and professional dental cleaning.

Since oxidative stress is reported to play a key role in both PCOS and PD, it may influence the bidirectional relationship between periodontal disease and PCOS, potentially exacerbating their inflammatory and metabolic consequences [21]. In this way, inflammasomes are key players in the innate immune response [22]; by driving excessive inflammation, tissue damage and bone resorption [23], they are the link between immune dysregulation and periodontal disease. The inflammasome is an intracellular multiprotein complex sensitive to both cellular stress signals and pathogens; it drives the autocatalytic activation of inflammatory caspases, ultimately leading to the release of mature cytokines, including interleukin 1β (IL1β) and interleukin 18 (IL18). The nucleotide-binding oligomerization domain (NOD), leucine-rich repeats (LRR) and pyrin domain-containing protein 3 (NLRP3) inflammasome are some of the most well-characterised inflammasomes, and their signalling pathway comprises coordinated two-step mechanisms consisting of priming and assembly. Priming upregulates NLRP3 and pro-IL1β via Nuclear Factor kappa-light-chain-enhancer of activated B cells (NFκB) activation through the Toll-like receptor (TLR) or cytokine signalling (e.g., Tumor Necrosis Factor alpha (TNFα), IL1β) [24,25]. Assembly is triggered by various pathogen-associated molecular patterns (PAMPs) (e.g., toxins, viral RNA) and damage-associated molecular patterns (DAMPs) (e.g., oxidised mitochondrial DNA, ATP), subsequently activating signalling pathways such as potassium efflux, lysosomal rupture and Reactive Oxigen Species (ROS) production [26,27,28,29]. This results in the formation of the NLRP3 complex, where NLRP3, Apoptosis-associated Speck-like protein containing a CARD (ASC) and procaspase-1 assemble into a multi-protein structure, leading to caspase-1 activation and IL1β maturation [30].

Accumulating evidence bestows NLRP3 inflammasome activation a central role in the pathogenesis of PD, especially periodontitis. In particular, lipopolysaccharide (LPS) from periodontal pathogenic bacteria is known to trigger the TLR4 signalling pathway, thus inducing the NLRP3 inflammasome signalling cascade [31]. Additionally, periodontal pathogens, such as *Porphyromonas gingivalis*, can provoke an NLRP3-dependent inflammatory response [32], while activation of the NLRP3 inflammasome is frequently detected in both the epithelium and connective tissue of the gingiva and its transcriptional levels are known to be elevated in the serum and saliva of PD patients [31,33]. However, the role of the NLRP3 inflammasome in leukocytes, which are in permanent circulation and capable of reflecting the metabolic and inflammatory status of the body [34], is poorly explored within the context of PD. We have previously reported hyperactivation of neutrophils in women with PCOS and have seen how it promotes their interaction with endothelial cells through exacerbated inflammatory and oxidative stress responses that are enhanced in the presence of gingivitis, suggesting that PCOS and PD are associated through inflammation processes and oxidative stress [35]. However, whether gingivitis is capable of modulating the inflammatory response mediated by the NLRP3 inflammasome in subjects with PCOS is an aspect that requires further exploration. Thus, the aim of the present study was to assess whether the presence of gingivitis is a relevant factor in the activation of the NLRP3 inflammasome in leukocytes of women with PCOS.

## 2. Materials and Methods

### 2.1. Study Design

This research is a case-control study carried out at University Hospital Doctor Peset in Valencia, Spain, between January 2019 and May 2022. The study population comprised women aged 18–45 years, including healthy individuals without PD and patients diagnosed with PCOS, both with and without gingivitis.

### 2.2. Participants

The PCOS group was identified according to the Rotterdam criteria, namely, irregular ovulation (menstrual cycles exceeding 35 days or under 26 days), elevated free testosterone levels (>0.5 ng/dL), hirsutism and polycystic ovaries. Gingivitis was diagnosed when bleeding on probing (BOP) was 10% or higher, following the latest consensus guidelines of various scientific societies specializing in PD [36]. Healthy women in the control group were matched for Body Mass Index (BMI) and age with those in the PCOS group.

Potential participants were excluded if they had other inflammatory conditions, cancer, bone-related diseases or periodontal conditions, diabetes or autoimmune disorders, and if there was recent antibiotic use or chronic anti-inflammatory medication. All the participants were informed in detail about the potential benefits and risks of the study, and all provided their written informed consent and signed a confidentiality agreement.

This human observational study was designed following STROBE (Strengthening the Reporting of Observational Studies in Epidemiology) guidelines and conducted in compliance with the Ethical Principles for Medical Research Involving Human Subjects outlined in the Declaration of Helsinki. All the procedures were approved by the Hospital Ethics Committee on April 24, 2019 (approval document Num. Ceim 31/19, registered under NCT06184412).

### 2.3. Anthropometric and Biochemical Determinants

The anthropometric and biochemical data of the cohort have been described in detail in Márquez-Arrico et al., 2024 [35].

### 2.4. Periodontal Parameters

We recorded the following periodontal measures: percentage of BOP; millimetres of clinical attachment level (CAL); millimetres of probing pocket depth (PPD); percentage of loss of bone; number of teeth with periodontal pockets—PPD ≥ 4 mm-; and number of teeth with CAL ≥ 4 mm-. We evaluated the plaque and calculus level using the Silness and Löe index for plaque [37] and the O’Leary index for calculus [38]. To simplify the measurement of plaque and calculus indices, they were assessed using the Ramfjord teeth index [39], which involves the measurement of six teeth per subject: the right permanent maxillary first molar, right permanent central incisor, left permanent first premolar, left permanent mandibular first molar, right permanent mandibular central incisor and right permanent mandibular first premolar.

### 2.5. Isolation of PBMCs

Peripheral blood mononuclear cells (PBMCs) were isolated in an EDTA-anticoagulated tube by means of the immunomagnetic method using a MACSprep leukocyte isolation kit (Milteny Biotech, Bergisch Gladbach, Germany), following the manufacturer’s instructions. In addition, the cell count and viability were assessed using LUNA-FL (Logos Biosystems, Annandale, VA, USA) through double staining with acridine orange and propidium iodide.

### 2.6. Flow Cytometry Assay

After erythrocyte lysis with RBC Lysis Solution (MACS, Milteny Biotech, Bergisch Gladbach, Germany), 200 μL of blood sample were labelled with 5 μL of APC-CD45 antibody for leukocytes identification (BD Biosciences, San Jose, CA, USA) and 1 mM Dihydroethidium (dHE) for superoxide determination (Invitrogen, Thermo Fisher Scientific, Waltham, MA, USA). Fluorescence was measured in a C6 Accuri cytometer (BD Biosciences, San Jose, CA, USA), and 10,000 events were analysed in each experiment.

### 2.7. Protein Expression Analysis

The PBMCs were lysed under ice-cold conditions for 15 min using the extraction buffer RIPA complemented with phosphatase inhibitors (Thermo Fisher Scientific, Waltham, MA, USA). The total protein concentration was quantified by the BCA method (Thermo Fisher Scientific, Waltham, MA, USA). A total of 25 μg of protein were separated by electrophoresis on a SDS–polyacrylamide gel and subsequently transferred to a nitrocellulose membrane. After blocking the membranes for 1 h using 5% skimmed milk, the proteins of interest were detected by overnight blotting at 4 °C with specific antibodies: mouse monoclonal anti-NFκB p65 (Invitrogen, Thermo Fisher Scientific, Waltham, MA, USA), rabbit polyclonal anti-JNK (Calbiochem, Merck Millipore, Darmstadt, Germany), rabbit monoclonal anti-NLRP3 (Cell Signaling Technology, Danvers, MA, USA), mouse monoclonal anti-ASC and anti-caspase-1 (Santa Cruz Biotechnology, Dallas, TX, USA). Mouse monoclonal anti-actin and rabbit polyclonal anti-actin were used as protein loading controls (Cell Signalling Technology, Danvers, MA, USA & Sigma-Aldrich, San Luis, MO, USA, respectively).

The following day, the membranes were incubated for 60 min at RT with secondary antibodies, namely, goat anti-rabbit (Vector Laboratories, Burlingame, CA, USA) and goat anti-mouse (Thermo Fisher Scientific, Waltham, MA, USA). The chemiluminescence signal was detected by adding ECL Plus reagent (GE Healthcare, Little Chalfont, UK) or SuperSignal™ West Femto (Thermo Fisher Scientific, Waltham, MA, USA). Visualisation was conducted using a Fusion FX5 Acquisition System and Cytiva Amersham ImageQuant 800, and signal quantification was performed using densitometry with Bio1D v15.03a software (Vilbert Lourmat, Marne-la-Vallée, France) and ImageQuant™ TL v10.2 analysis software (Cytiva, Marlborough, MA, USA).

### 2.8. RNA Extraction and Gene Expression Analysis

The total RNA was purified employing RibospinTM (GeneAll, Seoul, Republic of Korea), following the manufacturer’s instructions. The purity was assessed by the A260/A280 ratio obtained with a NanoDrop2000 (Thermo Fisher Scientific, Waltham, MA, USA). The mRNA expression level of the genes of interest was analysed by real-time PCR in a 7500 Fast Real-Time PCR System Instrument (Applied Biosystems, Foster City, CA, USA) using FastStart Universal SYBR Green Master (Rox) (Merck Millipore, Darmstadt, Germany). For each reaction, 5 ng cDNA were amplified with 0.20 µM of forward and reverse primers and master mix in a final volume of 10 µL. The sequences of the primer pairs used are available upon request. Following amplification, a melt curve analysis was performed. The relative gene expression was calculated with the 2^−∆∆Ct^ method, using cDNA obtained from the control subjects as a calibrator. *GADPH* (Glyceraldehyde Phosphate Dehydrogenase) and *18S* were used as reference genes, as their expressions were stable in the different conditions.

### 2.9. Evaluation of Systemic IL1β Levels

The serum levels of IL1β were measured in blood collected using gel serum separator tubes. They were centrifuged at 1500× *g* for 10 min at 4 °C and serum was immediately stored at −80 °C. Quantification was performed using a Luminex^®^ 200 analyser (Luminex Corporation, Austin, TX, USA), following the instructions provided by the MILLIPLEX^®^ kit manufacturer (Merck Millipore, Darmstadt, Germany). Samples with concentrations below the detection threshold were excluded from the analysis.

### 2.10. Statistical Analysis

The primary endpoint was the presence of NLRP3 inflammasome complex markers in a population of PCOS women with or without gingivitis. Given that there were 3 groups (including healthy controls), and assuming an alpha risk of 0.05 and a power of 90% in a two-tailed test, there needed to be 20 subjects in each group for a minimum difference of 35 units between any two groups to be statistically significant. The common deviation was assumed to be 30, and a drop-out rate of 0% was anticipated. Normal distribution of the samples was verified using the Kolmogorov–Smirnov or Shapiro–Wilk test. Data with normal distribution were presented as mean ± standard deviation (SD), while non-normally distributed data were shown as median (25th–75th percentiles). Qualitative data were represented as percentages. One-way ANOVA was employed to analyse the parametric data, and the Kruskal–Wallis test was used for non-parametric data. The association strength between the variables was assessed using Pearson’s or Spearman’s correlation coefficients. To predict the value of a variable based on another variable, linear regression analysis was employed. Differences were deemed significant when *p* < 0.05, with a 95% confidence interval. Data analysis was conducted using SPSS v22.0 (SPSS Statistics Inc., Chicago, IL, USA) and GraphPad Prism v9.0.2 software (GraphPad Software, San Diego, CA, USA).

## 3. Results

### 3.1. Study Population and Group Characteristics

A total of 36 healthy control females without PD or clinical/biochemical signs of PCOS or other metabolic diseases were recruited for this study, along with 68 women with PCOS, including 44 without PD (PCOS group) and 24 with gingivitis (PCOS+ group). The anthropometric and biochemical data have been published previously [35]. Age and BMI were similar among the three groups, while levels of high-sensitivity C-reactive protein (hsCRP) (0.81 (0.34, 3.10) vs. 3.16 (0.66, 5.32), *p* < 0.05) and TNFα (9.6 (7.5, 13.7) vs. 13.3 (9.9, 15.7) (*p* < 0.05)) were higher in the PCOS population versus the controls, but not significantly different between the PCOS and PCOS+ groups. Understandably, the values of the periodontal parameters BOP, plaque index and calculus index were greater in the PCOS+ group (Table 1), while no significant differences in the remaining periodontal parameters (PPD, CAL, tooth loss and periodontal treatment) were observed among the groups, which is in line with data recently published by our group [35].

### 3.2. Proinflammatory Mediators

**Figure 1:**
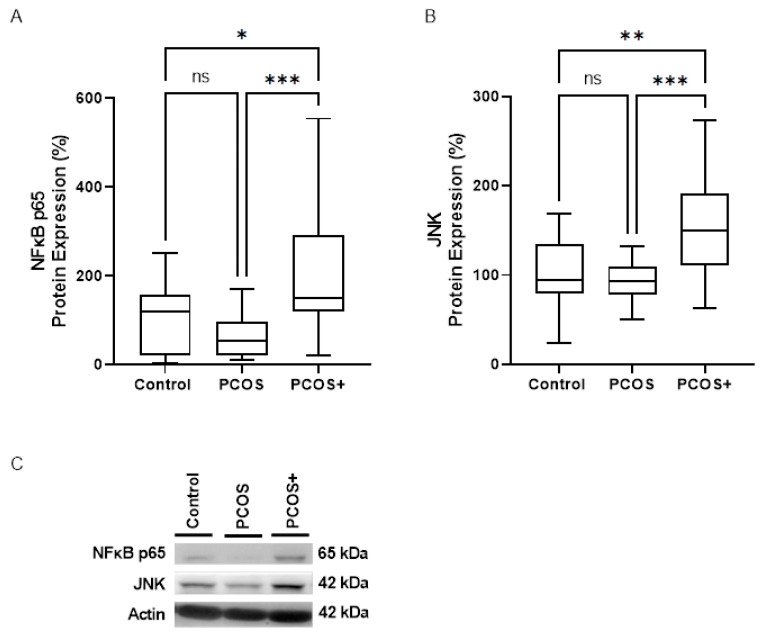
Protein levels of proinflammatory master regulators in PBMCs from control subjects and women with PCOS with and without gingivitis. Images show protein levels relative to the actin signal for NFκB-p65 (**A**) and JNK (**B**), along with images from the Western blot experiments (**C**). Data are presented as box-and-whisker plots. ns, statistically non-significant, * *p* < 0.05; ** *p* < 0.01; *** *p* < 0.001 when the three groups were compared by means of a Kruskal–Wallis with Dunn’s post hoc test., JNK, Jun N-terminal kinase; NFκB p65, nuclear factor kappa-light-chain-enhancer of activated B cells; PBMCs: Peripheral Blood Mononuclear Cells; PCOS, polycystic ovary syndrome; PCOS+, polycystic ovary syndrome with gingivitis.

Since PCOS is associated with chronic systemic inflammation, we aimed to evaluate the primary mediators of this response in PBMCs from the whole cohort. A Western blot analysis revealed a significant increase in the relative protein levels of the classic inflammatory pathways NFκB and JNK (Figure 1) in the PCOS+ versus the PCOS group, but we did not detect any changes between the healthy and PCOS subjects without PD These results suggest that gingivitis was instrumental in inducing the priming step of NLRP3 inflammasome formation in patients in the PCOS+ group, thus indicating an enhanced inflammatory status among these individuals (Figure 1).

### 3.3. NLRP3 Inflammasome Assembly Engagement

**Figure 2:**
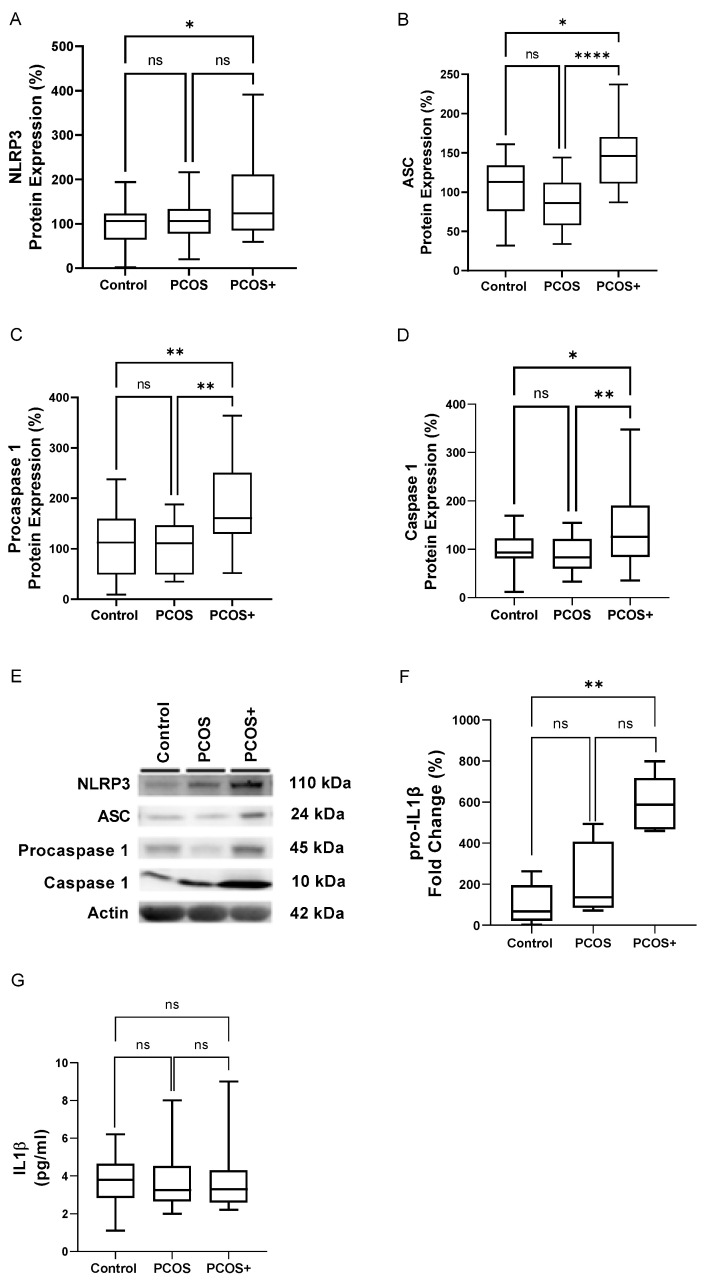
Protein expression of NLRP3 inflammasome-related proteins, mRNA levels of *pro-IL1β* in PBMCs and serum levels of the proinflammatory cytokine IL1β from control subjects, PCOS and PCOS+ subjects. Protein levels of NLRP3 (**A**), ASC (**B**), procaspase-1 (**C**) and caspase-1 (**D**) are normalised to the loading control actin, with corresponding Western blot images, levels of mRNA of *pro-IL1β* and serum IL1β levels represented in (**E**–**G**), respectively. Data are displayed as box-and-whisker plots. ns, statistically non-significant, * *p* < 0.05; ** *p* < 0.01; **** *p* < 0.0001, as determined by a Kruskal–Wallis test with Dunn’s post hoc comparison of the three groups. ASC: Apoptosis-associated Speck-like protein containing a CARD; NLRP3: nucleotide-binding oligomerization domain (NOD), leucine-rich repeats (LRR) and pyrin domain-containing protein 3; IL1β: interleukin 1β; PCOS: polycystic ovary syndrome; PCOS+: polycystic ovary syndrome with gingivitis.

To confirm that the NLRP3 inflammasome had formed, the relative levels of its components were determined. Our results show a significant increase (*p* < 0.05) in the protein levels of NLRP3 (Figure 2A), ASC (Figure 2B), procaspase-1 (Figure 2C) and caspase-1 (Figure 2D) and in the *pro-IL1β* mRNA levels (Figure 2E) in subjects with both PCOS and gingivitis, while no differences were detected in the serum IL1β levels (Figure 2G). These findings suggest that gingival inflammation modulates inflammatory pathways in PBMCs, including the NLRP3 inflammasome, by enhancing the expression of its core components.

### 3.4. Oxidative Stress and Antioxidant Response

**Figure 3:**
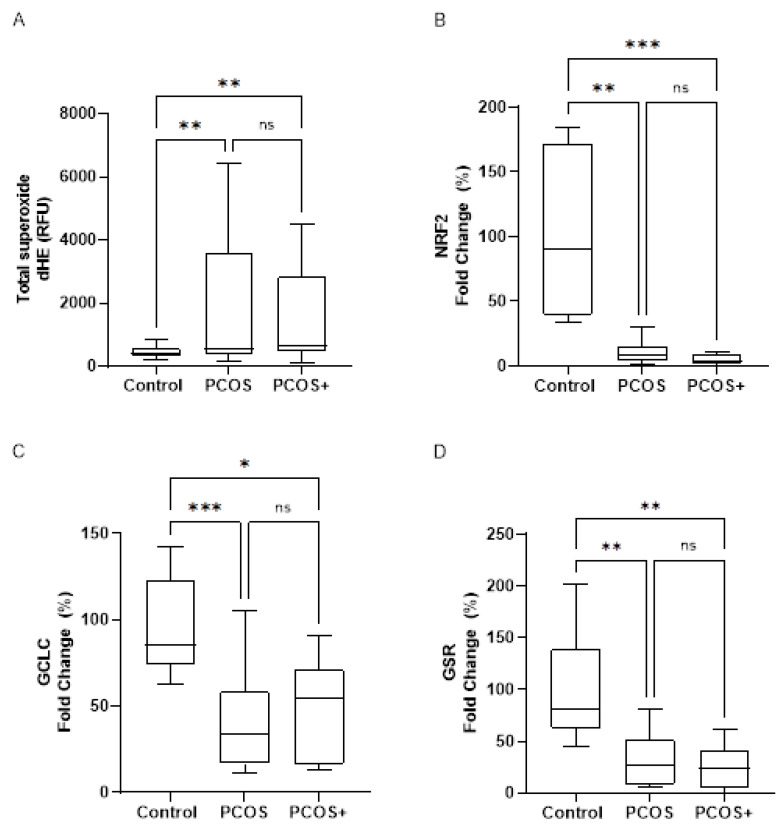
Oxidative stress parameters in PBMCs from patients with PCOS with and without gingivitis and control subjects. Total superoxide levels (**A**) are represented as the median fluorescence intensity of dHE, and mRNA levels of *NRF2* (**B**) and *NRF2*-target genes *GCLC* (**C**) and *GSR* (**D**) are normalised with respect to control patients. Data are presented as box-and-whisker plots. ns, statistically non-significant, * *p* < 0.05; ** *p* < 0.01; *** *p* < 0.001 when the three groups were compared by means of a Kruskal–Wallis with Dunn’s post hoc test or one-way ANOVA with Student–Newman–Keuls as a post hoc test. *GCLC*: Glutamate–Cysteine Ligase Catalytic subunit; *GSR*: Glutathione Reductase; dHE: Dihydroethidium; *NRF2*: Nuclear factor erythroid 2–related factor 2; PBMCs: Peripheral Blood Mononuclear Cells; PCOS: polycystic ovary syndrome; PCOS+: polycystic ovary syndrome with gingivitis; RFU: Relative Fluorescence Units.

In light of the aforementioned results, we explored the influence of oxidative stress on the activation of the NLRP3 inflammasome complex in PBMCs, since ROS levels are related to inflammasome activation. Our results show that the total superoxide levels were increased in the women with PCOS but did not differ between the PCOS and PCOS+ group (Figure 3A), thus highlighting the involvement of ROS in PCOS. Given the increase in pro-oxidants in the PCOS group, we explored the state of the patients’ antioxidant defences by activating the Nuclear factor erythroid 2–related factor 2 (*NRF2*)-derived antioxidant response. The mRNA levels of *NRF2* (Figure 3B), as well as Glutamate–Cysteine Ligase Catalytic subunit (*GCLC*) (Figure 3C) and Glutathione Reductase (*GSR*) (Figure 3D), were lower in the PCOS group, demonstrating that the *NRF2*-derived antioxidant response was impaired among our women with PCOS.

### 3.5. Correlations Between Gingival Inflammation and Molecular Markers

**Figure 4:**
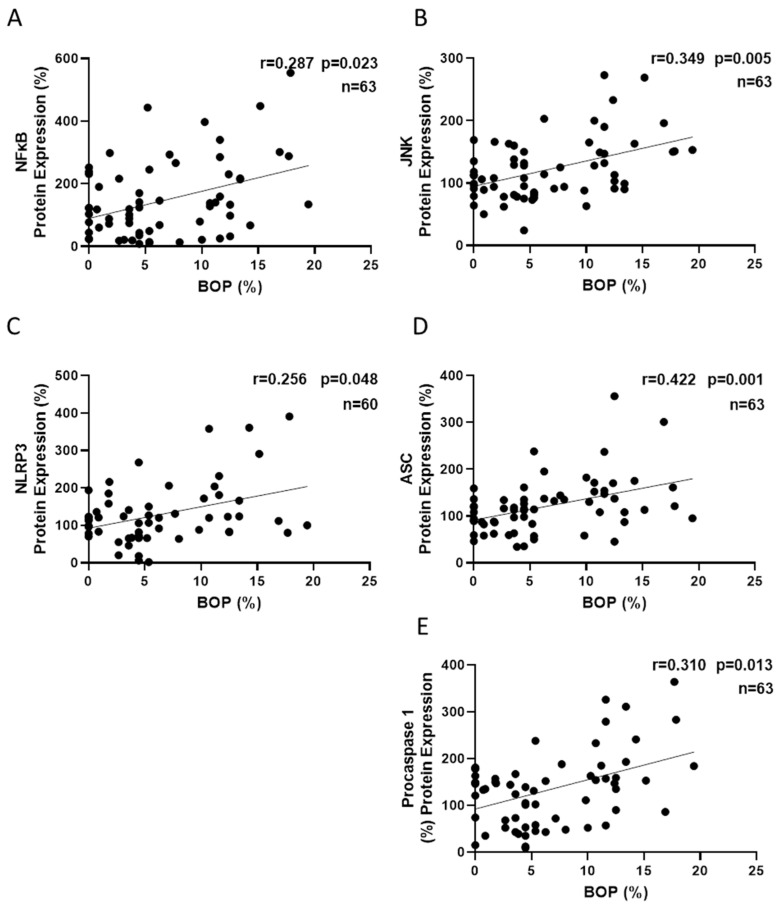
Correlations between levels of inflammatory markers and clinical parameters of gingivitis BOP. NFκB (**A**), JNK (**B**), NLRP3 (**C**), ASC (**D**) and procaspase-1 (**E**) correlated positively with BOP (*p* < 0.05). ASC: Apoptosis-associated Speck-like protein containing a CARD; BOP, bleeding on probing; JNK, Jun N-terminal kinase; NFκB p65, nuclear factor kappa-light-chain-enhancer of activated B cells; NLRP3: nucleotide-binding oligomerization domain (NOD), leucine-rich repeats (LRR) and pyrin domain-containing protein 3; PCOS, polycystic ovary syndrome.

Finally, when we explored potential correlations among the BOP and inflammatory mediators, we found that BOP correlated positively with NFκB (r = 0.287; *p* = 0.023), JNK (r = 0.349; *p* = 0.005), NLRP3 (r = 0.256; *p* = 0.048), ASC (r = 0.422; *p* = 0.001) and procaspase-1 (r= 0.310: *p* = 0.013) (Figure 4). However, none of the oxidative stress parameters (superoxide, *NRF2*, *GCLC* and *GSR*) correlated with BOP.

In order to further explore the association between BOP and the correlated variables, we carried out a multivariable regression model using the stepwise method (Table 2).

Our results show that JNK (β = 0.275), ASC (β = 0.297) and procaspase-1 (β = 0.284) were independently associated with BOP, explaining 34.8% of the dependent variable (*p* < 0.001).

## 4. Discussion

In the present study, we demonstrate that gingival inflammation activates the innate immune response through modulation of the NLRP3 inflammasome complex in the PBMCs of PCOS patients. This response is regulated by proinflammatory transcriptional factors and oxidative damage, which are involved in the priming and assembly of the NLRP3 inflammasome, respectively.

It is well established that an imbalance of dental microorganisms plays a key role in the onset and progression of PD, including reversible conditions, such as gingivitis [18,40,41]. Plaque-induced gingivitis is caused by an alteration of subgingival microbiota composition. This dysbiosis promotes excessive growth of pathogenic species, which triggers an innate immune response in gingival tissue [20,40]. Additionally, intracellular innate immune sensors, such as the NLRP3 inflammasome, respond to bacterial challenge, initiating early host responses [22,30,42]. In fact, different studies have reported increased mRNA and protein levels of NLRP3 in gingival tissues and saliva from patients with PD, especially those with periodontitis [31,33,43], suggesting there is local priming of the NLRP3 inflammasome in PD.

Furthermore, it is well documented that LPS from pathogenic bacteria can trigger TLR4 activation, which, in turn, activates NFκB and leads to the expression of the NLRP3 protein and cytokines such as pro-IL1β at the end of the priming stage [44]. In line with such previous research, our present findings highlight an increase in the inflammatory mediator NFκB in the PBMCs from patients with PCOS and gingivitis, along with a rise in JNK levels, whose induction of NLRP3-phosphorylation has been reported as a critical and essential priming event for NLRP3 self-association and subsequent inflammasome activation [45], which was aligned with an increase in *pro-IL1β* mRNA levels. This suggests that gingivitis triggers the priming stage of the NLRP3 inflammasome, probably due to the presence of periodontopathogenic bacteria. This is supported by the positive correlation we detected between gingival inflammation (BOP) and both NFκB and JNK, and by the role of JNK in our multivariable independent regression model.

Following completion of the priming signal, assembly signals, such as DAMPs and PAMPs, trigger the formation of the NLRP3 complex through the assembly of NLRP3, ASC and procaspase-1. In this context, Isola G. et al. described higher levels of NLRP3 in the serum of patients with periodontitis [33]. During the experiments described herein, we specifically observed higher levels of NLRP3, ASC, procaspase-1 and caspase-1 in the PBMCs from PCOS+ patients, suggesting once again that gingival inflammation plays a pivotal role in the process of NLRP3 complex assembly, as indicated by our correlation analyses and a multivariate regression model. NLRP3 inflammasome activation has been reported in the ovaries of hyperandrogenism-induced rodents [46] and in ovarian granulosa cells from women with PCOS [47], suggesting localised inflammation in this condition. Our findings provide strong evidence of priming during PD, suggesting assembly and activation of the NLRP3 inflammasome at a systemic level, though we did not detect significant differences in systemic IL1β. It is important to note that systemic IL1β levels are regulated by multiple cell types, including monocytes, macrophages, neutrophils and endothelial and epithelial cells. As a result, the overall concentration of IL1β is the result of a complex interplay among these cell populations. Therefore, the absence of significant changes in systemic IL1β levels in our population may have been related to the heterogeneous contributions of these cells, whose individual roles in this process are still not fully understood [48].

Oxidative stress can trigger inflammatory responses by activating key signalling processes, such as NLRP3 inflammasome assembly [49], while also stimulating NRF2 activation, which, in turn, induces the expression of antioxidant response genes that protect the cell from oxidative insults [50]. In the present study, we observed higher levels of superoxide in the PBMCs from the subjects with PCOS compared to those from the controls, which is consistent with previous findings [50,51,52,53], though differences were not detected between the PCOS and PCOS+ groups. In a similar sense, Almerich-Silla et al. did not observe significant differences in the oxidative stress parameters 8-OHdG, GPx, TAOC, SOD and MDA in saliva from patients with gingivitis versus counterparts without PD, which is in line with our findings [19].

Similarly, we observed that the expression of *NRF2* and *NRF2*-dependent antioxidant response genes was lower in PCOS-derived PBMCs, suggesting an unbalanced redox state related to PCOS. Such redox impairment has previously been described in the physiopathology of PCOS and has been associated with IR [54]. On the other hand, several studies regarding periodontitis have pointed to *NRF2* impairment as a key factor in oxidative stress-associated periodontal damage [55]. For instance, Sima et al. observed a decrease in *NRF2* expression in oral and blood polymorphonuclear cells from patients with severe chronic periodontitis [56], suggesting that oxidative stress plays a prominent role in advanced periodontal disease. However, we did not detect such an association during our study. Since gingivitis is an early, mild and reversible PD, we suspect that the oxidative damage it produces in PBMCs does not manifest itself, in contrast to that occurring in periodontitis [19,57]. Since ROS is known to trigger the assembly of the NLRP3 inflammasome complex [58], and given that we observed priming exclusively in the PBMCs from our PCOS+ patients, we suspect that assembly was initiated in patients with gingivitis, particularly because we observed maturation of procaspase-1 into caspase-1. Hence, our findings would indicate that NLRP3 inflammasome activation is related to the presence of LPS and toxins generated by periodontopathogenic bacteria in women with PCOS who have previously been in a state of oxidative stress that drives the progression of the inflammatory process.

As far as we know, this study provides initial insight into NLRP3 inflammasome activation at the systemic level in the context of gingivitis. Our findings complement previous local-focused studies reporting higher levels of NLRP3 in the gingival tissue of periodontitis patients. This study has some limitations, including the relatively small sample of women with gingivitis, though it was supported by sample size calculation. Moreover, the cross-sectional nature of the study limits its interpretability. Future research should address these issues.

In summary, our results shed light on the systemic effects of gingivitis, establishing a new focus for periodontal research investigating inflammation associated with biofilm-derived gingivitis, an area as yet not well explored. Further research should seek to determine the role of NRLP3 in the early stages of inflammatory diseases, such as gingivitis, in the context of PCOS.

## 5. Conclusions

Our findings provide novel insight indicating that, within a proinflammatory and pro-oxidant context, gingivitis can activate the innate immune system, leading to an exacerbated inflammatory response. This is particularly relevant in women with PCOS, a condition characterised per se by chronic low-grade inflammation and elevated oxidative stress, which can further potentiate systemic inflammatory responses when exacerbated by PD. Given the well-established associations between oxidative stress, metabolic dysfunction and reproductive disturbances in PCOS, periodontal inflammation may constitute an additional pathogenic stimulus, exacerbating IR, altering endocrine homeostasis and potentially increasing the risk of cardiovascular disease. These findings not only shed light on the bidirectional relationship between PD and systemic inflammatory conditions like PCOS but also highlight the broader systemic consequences of an untreated periodontal pathology. Maintaining a plaque-free status remains challenging due to anatomical complexities and inconsistent patient adherence. Nevertheless, considering its high prevalence and potential impact on systemic health, the effective management of gingivitis is crucial. This underscores the urgent need for innovative therapeutic approaches aimed at addressing local and systemic inflammatory processes in order to mitigate the progression of PCOS-related complications and improve the overall health outcomes of this pathology.

## Figures and Tables

**Table 1:** Periodontal parameters according to each group.

	Control	PCOS	PCOS+
BOP (%)	1.935 ± 2.336	3.211 ± 2.644	13.162 ± 4.184 *#
Plaque index	0.423 ± 0.408	0.598 ± 0.472	0.843 ± 0.608 *#
Calculus index	0.021 ± 0.053	0.022 ± 0.066	0.165 ± 0.436 *#

Data are presented as mean ± standard deviation. A one-way ANOVA was used for analysis, followed by a Student–Newman–Keuls post hoc test. Values with different superscript symbols indicate significant differences (*p* < 0.05) between three groups: * denotes a significant difference compared to the Control group, and # denotes a significant difference compared to the PCOS group. BOP, bleeding on probing; PCOS, polycystic ovary syndrome; PCOS+, polycystic ovary syndrome with gingivitis.

**Table 2:** Multivariable linear regression model of parameters of NLRP3 inflammasome-related proteins as dependent variables.

Dependent Variable	Independent Variable	B	Standard Error	Beta Coefficient	Adjust R2	*p*-Value
BOP					0.348	<0.001
	JNK	0.030	0.012	0.275		0.012
	ASC	0.027	0.010	0.297		<0.001
	Procaspase-1	0.020	0.007	0.284		<0.001

ASC: Apoptosis-associated Speck-like protein containing a CARD; BOP: bleeding on probing; JNK: Jun N-terminal kinase; NLRP3: nucleotide-binding oligomerization domain (NOD), leucine-rich repeats (LRR) and pyrin domain-containing protein 3.

## Data Availability

Data are available upon reasonable request.

## Abbreviations

The following abbreviations are used in this manuscript:

ASC	Apoptosis-associated Speck-like protein containing a CARD
BOP	Bleeding on probing
BMI	Body Mass Index
CAL	Clinical Attachment Level
DAMPs	Damage-associated molecular patterns
dHE	Dihydroethidium
*GCLC*	Glutamate–Cysteine Ligase Catalytic subunit
*GSR*	Glutathione Reductase
hsCRP	High-sensitivity C-reactive protein
IL1β	Interleukin 1β
IR	Insulin resistance
JNK	c-Jun N-terminal kinase
LPS	Lipopolysaccharide
NFκB	Nuclear Factor kappa-light-chain-enhancer of activated B cells
NLRP3	Nucleotide-binding oligomerization domain (NOD), leucine-rich repeats (LRR) and pyrin domain-containing protein 3
*NRF2*	Nuclear factor erythroid 2–related factor 2
PAMPs	Pathogen-associated molecular patterns
PBMCs	Peripheral blood mononuclear cells
PCOS	Polycystic ovary syndrome
PD	Periodontal disease
PPD	Probing Pockets Depth
ROS	Reactive Oxygen Species
TLR	Toll-like receptor
TNFα	Tumor necrosis factor alpha
